# A Database of Static Thermal Insulation and Evaporative Resistance Values of Dutch Firefighter Clothing Items and Ensembles

**DOI:** 10.3390/biology11121813

**Published:** 2022-12-13

**Authors:** Kalev Kuklane, Jakob Eggeling, Maurice Kemmeren, Ronald Heus

**Affiliations:** 1 Team Fire Service Science, Netherlands Academy of Crisis Management and Fire Service Science, Netherlands Institute for Public Safety, Zilverstraat 91, 2718 RP Zoetermeer, The Netherlands; ronald.heus@nipv.nl; 2 Division of Ergonomics and Aerosol Technology, Department of Design Sciences, Lund University, Box 118, 22100 Lund, Sweden; jakob.eggeling@design.lth.se; 3 Team COLS, Netherlands Institute for Public Safety, Zilverstraat 91, 2718 RP Zoetermeer, The Netherlands; maurice.kemmeren@nipv.nl

**Keywords:** clothing system, firefighter, station wear, turnout gear, clothing area factor, local and regional thermal insulation, local and regional evaporative resistance, modelling

## Abstract

**Simple Summary:**

Rescue services personnel may be exposed to a wide range of harsh environments that require the use of protective gear. In order to achieve the best performance, the optimal protection for specific incident scenarios should be selected. In order to be able to specify the protective clothing ensemble that best matches the protection needs, activity and environmental conditions, while keeping up the performance and reduce impact of thermal stress, we need to know the clothing properties that affect heat transfer via the protective layers. The thermal insulation and evaporative resistance values of firefighter clothing items and ensembles of the whole protective system are not easily available. The work presented in this paper fills this gap. Collected data allow for validation and selection of prediction models for exposure evaluation, and with this, it contributes to the best performing protective ensemble choice for specific incident scenarios. The paper also presents the relationships for summing individual clothing items’ insulation of the firefighter protective clothing system and therefore reduces the need for separate testing of all possible clothing ensemble configurations. The wide literature background allows for extrapolation of the results to other branches where protective clothing is used.

**Abstract:**

The rescue operations’ environment can impair firefighters’ performance and increase the risk of injuries, e.g., burns and hyperthermia. The bulk and carried weight of heavy protection contributes to lower physical performance, higher metabolic load and internal body heat production. For recommending optimal protection for the tasks and incident scenarios, knowledge of clothing thermal properties is needed. However, detailed data on firefighter protective clothing systems are not available. The aim of the study was to provide scientific background and a dataset that would allow for validation of thermo-physiological models for task-specific conditions of rescue work. Thermal insulation of 37 single items and their variations and 25 realistic protective clothing ensembles were measured on a thermal manikin. Twelve (12) ensembles that evenly covered the whole insulation range were selected for evaporative resistance testing. The equations for summing up individual item’s insulation to ensemble insulation and calculating clothing area factor were derived from the dataset. The database of a firefighter clothing system was created. In addition, the local and regional thermal properties of the clothing ensembles were provided for use in future validation of advanced thermo-physiological models for rescue worker exposure predictions and for designing decision aid tools.

## 1. Introduction

During rescue operations, the environment can impair firefighters’ performance and increase the risk of injuries such as burns [1,2] and hyperthermia [3,4,5]. The firefighter ensembles offer high protection levels. However, increased protection also increases the bulk and carried weight that means lower physical performance, higher metabolic load and internal body heat production [6,7,8]. Reduced mobility, flexibility, and dexterity may also restrict the ability to successfully accomplish a task [9,10,11,12]. Therefore, an objective aim of firefighter organizations is being able to select optimal protection for the tasks and incident scenarios.

In the Netherlands, the fire services are currently switching to a new station wear, called operational uniform (OU) for low-risk tasks. It is very important to look at how firefighters can in the future be optimally protected within the entire risk spectrum of both low-risk and high-risk tasks. A current limitation is an insufficient database and knowledge about the thermal properties of firefighter protective clothing items and systems. There is currently no coordination of (protective) clothing packages in use for operational tasks. At every alarm, the protective clothing for firefighting is put on, with or without the current station uniform, or in the future, on the operational uniform. For volunteer firefighters, often, only the protective clothing for firefighting is available, and this is worn over their daily clothing. Risk profiles, protection requirements and wearing comfort play an important role in order to arrive at the protection concept that is best suited to the tasks. It is desirable that such a protection concept leaves space for integration of future developments.

With regard to functionality and protection, the minimum level has been established and formalized by means of (inter)national standards (NEN-EN and ISO) [13,14,15,16,17,18,19,20,21,22]. However, most of the requirements are related to materials and material packages and not for the complete items or clothing ensembles. The current clothing systems in use are combined where possible and should ultimately lead to a fully integrated protective clothing system, of which digital support can be a part, for example, mobile applications, such as ClimApp [23,24] and decision aids [25,26]. In the future, possibly, the clothing system can be combined with the necessary sensors and smart solutions to acquire real-time input from the incident site to allow for the optimal selection of gear. Firefighters have to be adequately protected for the tasks they have to perform, whereby each task may require specific protection. An aim is that the clothing can be built up by modules into an integral protection concept. Modularity in clothing systems may also support sustainable development with easier inspection, care and maintenance of the gear, allowing for replacement of worn out items and longer lasting of the whole protective clothing system.

Clothing insulation and evaporative resistance are the basic clothing parameters that affect human heat exchange with the environment. They are used as a common behavioural thermoregulatory measures and are means to maintain thermal comfort in a wide range of temperatures [27]. Commonly, these properties are not evenly distributed over the whole body surface due to the material choice, clothing design, layering, fit, etc. Physiological parameters, such as heat generation in muscles of different body parts during different work tasks, body internal heat distribution and areas for heat loss [28,29], protection criteria of vital regions, sweating patterns [30,31,32], but also freedom of motion, may set the specific demands on the need of regional evaporative resistance [33] and clothing insulation [34]. Clothing parameters for body regions can be measured, and the change due to walking and wind can be estimated [35,36,37,38]. Local values can be used in advanced physiological models for exposure evaluation [26,39,40,41,42,43,44], for occupational health and safety purposes, as a feedback for clothing manufacturers for improving their products, and/or for industries for selecting the clothing provided to their employees. In addition, some publications provide information on how to count for fit, garment size and/or airgaps [37,43,45,46].

The methods to measure or estimate clothing items’ or their combinations’ insulation [47,48,49,50] and evaporative resistance [51,52] are standardized, or detailed suggestions for testing and data treatment are available [53]. ISO 9920 [50] contains databases on clothing items and ensembles based on earlier studies [54,55,56], and presents algorithms to sum individual items, calculate effect of wind and motion, etc. Despite the extensive content of this standard [50], it has a limited coverage of different clothing styles other than Western office and workwear, while recent publications cover that gap [57,58,59]. Another problem is that the majority of the standard’s database was compiled mainly around 1980s, and both the design and the materials have changed. This has now been overcome by many recent studies on modern office and casual clothing that have been measured using the latest technology and providing detailed information on different aspects of clothing thermal performance [36,37,38,39,42,43,60,61,62,63]. At the same time, when modern office and casual clothing are measured in a systematic way, data on industrial workwear are not as easily available. Some exceptions are studies on military [64,65,66,67,68,69], ambulance [70,71], cold protective [72,73,74,75,76,77], and some agricultural [78,79] clothing systems. Although, the studied clothing systems cover a wide variety of clothing configurations, it may be suspected that specialized clothing, e.g., the ones used by firefighters, may behave differently due to the protective layers and their combinations. Considering any future developments in clothing science and advanced support systems [23,25,26], and to select the best suited protection for any incident scenario in high stress situations quickly, more detailed information on thermal properties of clothing that is used by rescue workers is needed. As it is quite impossible to measure all possible clothing combinations used in the field, it is important to create an individual clothing items’ wardrobe database based on manikin measurements. Development and/or validation of specific clothing insulation and evaporative resistance summation methods that match the selected clothing system would allow for evaluation of human exposure and to select the proper protection over a wide range of environments and working conditions [23,26,40,78,80,81,82,83,84,85].

**Figure A1:**
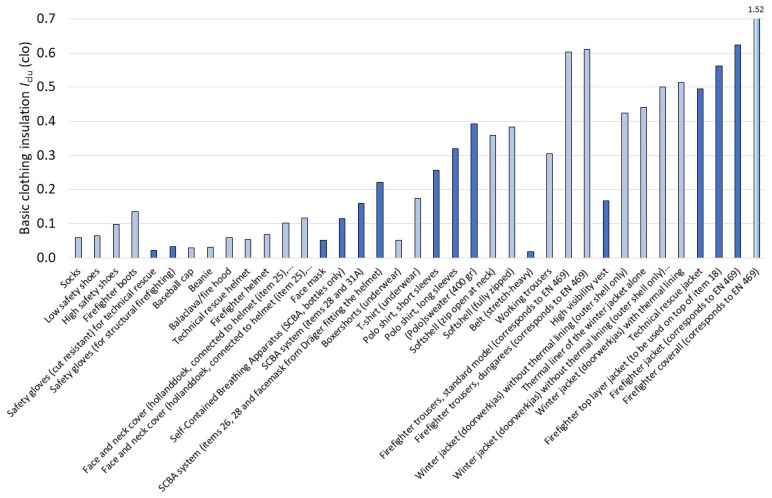
Basic clothing insulation of the tested items (*I*_clu_, clo). As the firefighter coverall had over 2 times higher basic insulation, the *y*-axis scale was not extended to that level, but the value (1.52 clo) is given above the top of this column. Alternating colour shade groups differentiate between product groups and/or body regions covered by these items, e.g., feet (socks, shoes), hands (gloves) head, SCBA element variations, etc.

**Figure A2:**
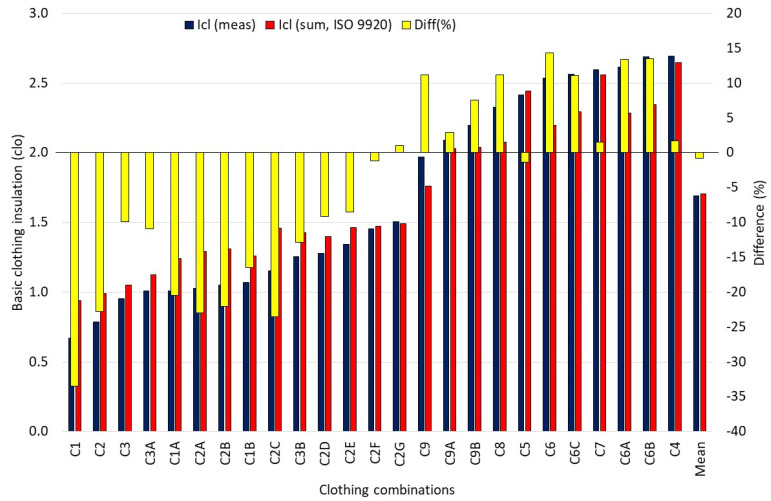
Measured (Icl (meas)) and summed (Icl (sum, ISO 9920)) basic clothing insulation of all ensembles (*I*_cl_, clo) with their relative differences (Diff(%)). For clothing combination descriptions see Table 1, Table 2 and Table 3.

**Figure A3:**
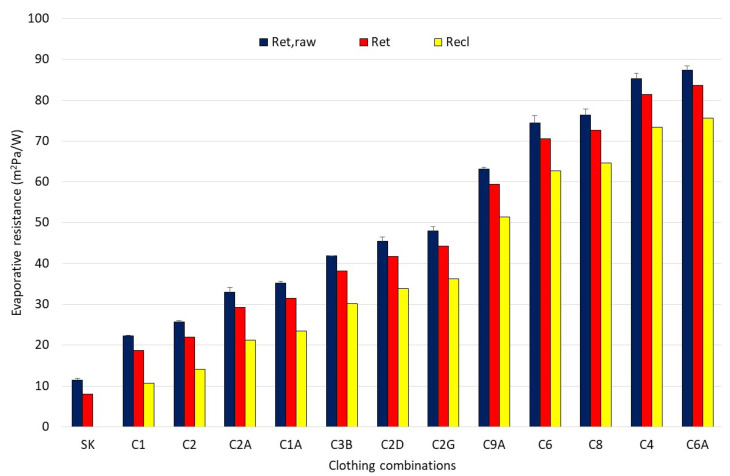
Uncorrected total evaporative resistances (Ret,raw), and according to Wang et al. [98], corrected total (Ret) and clothing (Recl) evaporative resistances of the tested clothing ensembles (m^2^Pa/W). For clothing combination descriptions, see Table 1, Table 2 and Table 3.

This work aimed to create a detailed firefighter clothing database of static thermal insulation values of the individual clothing items, some PPE items, and their combinations that are the elements of Dutch firefighter station wear (operational uniform) and incident scenario-based turnout gear. An aim was also to report the static thermal insulation of realistic clothing ensembles of station wear and turnout gear and evaporative resistance of selected clothing ensembles. As the collected material is quite extensive, local insulation and evaporative resistance values of the clothing ensembles for various body regions that may be useful for advanced thermo-physiological modelling are included in Appendix A and Appendix B (Table A1 and Table A2, respectively). The items’ and ensembles’ thermal properties are ranked and visualized in Appendix C (Figure A1, Figure A2 and Figure A3).

## 2. Materials and Methods

### 2.1. The Thermal Manikin Tore

In this study, all items were tested individually or in combinations on the thermal manikin Tore at the Thermal Environment Laboratory, Lund University. Tore is a male-shaped thermal manikin [86,87]. Tore is made of orthopaedic foam with a metal frame inside to support the body parts and for joints. Tore is divided into 17 individually controlled zones: head, chest, back, stomach, buttocks, left and right upper arm, left and right lower arm, left and right hand, left and right thigh, left and right calf, and left and right foot. The weight of the manikin is 32 kg. The surface temperature of the manikin’s zones was controlled at 34 °C. Thermal manikin Tore and the used climatic chamber have been used in multiple joint studies and interlaboratory trials, and comparative data with the other manikins and test environments are well available over the wide range of clothing types, postures, walking and wind velocities, and for the variety of purposes, e.g., for thermal insulation [58,63,72,88], evaporative resistance [89], for thermo-physiological modelling [90], and as a part of evaluation of occupational incident [91], cooling systems [92,93], etc.

### 2.2. Manikin Calibration

Offset calibration of the manikin’s surface temperature sensors was carried out in the homogenous conditions (34 °C) in a warm chamber with the same Pico equipment that was used for air temperature measurements, as described in Section 2.4. Calibration procedures followed ISO 15831 [49].

### 2.3. Measurements of Clothing Surface Area Factor (f_cl_)

Photographic method based on 2 photos was used to estimate clothing area factor (*f*_cl_) of the individual garments and the ensembles [52,58]. The frontal and the side photos were used.

### 2.4. Thermal Insulation Measurements

The tests for total thermal insulation (*I*_T_) were carried out on the manikin in a standing position according to ISO 15831 [49] following ISO 9920 [50] recommendations (low air velocity). The basic (intrinsic) insulation of each garment item (*I*_clu_) and clothing ensemble (*I*_cl_) was calculated. The mean air velocity was measured with Swema Air 3000d logger with omnidirectional SWA03 sensor 7 Air velocity in the chamber stayed on average at 0.18 ± 0.07 m/s. It was originally measured as an average of 15 points in air flow 50 cm before the manikin and then checked in the control position at the end of each individual test. Air layer insulation (*I*_a_) was measured with a nude manikin in the same conditions. It has been shown that the calculation method (parallel or serial) may strongly affect the result [76,94,95,96,97]. When testing highly insulative and less permeable clothing, the manikin heat loss from some zones, especially in the areas with many overlapping layers, e.g., buttocks, abdomen, may drop close to zero, and local temperature may rise above the set temperature. Therefore, the principles of global method [76,97] that are insensitive for such technical effects and are also more reliable for correlating with human exposures, were utilized in the calculations.

The air temperature in the climate chamber was set to 20 °C for individual items and light clothing combinations and to 10 °C for heavy protective combinations. Air temperature variation during the stable state was below 0.1 °C. Ambient air temperature was continuously monitored using three sensors (PT 100, Pico Technology Ltd., St. Neots, UK) positioned adjacent to the ankles, the mid-trunk, and the head (vertical heights of 0.1, 1.1, and 1.7 m from the soles of the manikin). The mean radiant temperature was equal to the air temperature. The temperatures and heat losses were recorded at ten-second intervals. The last 10 min of stable state was used for thermal insulation calculation.

### 2.5. Evaporative Resistance Measurements

The total evaporative resistance (*R*_et_) of selected clothing ensembles was measured according to ASTM F2370-16 [51] on the manikin in standing position under so-called isothermal conditions set to 34 °C. Both the mass loss and heat loss methods were used for further analysis, but as comparable local evaporative resistances of body regions could only be based on heat loss, only the results from the heat loss method are presented in this paper. Average air velocity during these tests was 0.40 ± 0.13 m/s. Data for the whole measuring period (commonly up to 70 min) were saved. Based on the weight change, temperature and heat loss curves, the stable periods, meaning constant evaporative heat loss, were selected for evaporative resistance calculation. Relative humidity was measured with the Humidity Sensor Evaluation Kit EK-H3 with pin SHT75 sensors (Sensirion AG, Stäfa, Switzerland). Relative humidity in the chamber was set to 40%. During the stable state of each measurement, the variation in the relative humidity was less than 2%. The corrections according to Wang et al. [98] were applied to the measured evaporative resistance values. Any further corrections, e.g., for skin material and moist skin insulation, etc. [52,53,99,100,101], were not used. The further comparisons between different available corrections [53,102] will be presented in a separate paper. Finally, clothing evaporative resistance (*R*_ecl_) and permeability indices (*i*_m_ and *i*_m,cl_) were calculated.

### 2.6. Calculations

Total insulation of an individual zone (*I*_T,i_, m^2^K/W):

(1) $$I_{T,i}=\frac{\left(T_{s,i}-T_{o}\right)\times A_{i}}{H_{c,i}},$$

where *T*_s,i_—mean surface temperature of a manikin zone *i* (°C); *T*_o_—operative temperature (°C); *A*_i_—area of a manikin zone *i* (m^2^); *H*_c,i_—dry heat loss from a manikin zone *i* (W).

An individual zone insulation as a contribution to the whole body insulation (*I*_T,i,r_, m^2^K/W):

(2) $$I_{T,i,r}=\frac{A_{i}}{A}\times \frac{\left(T_{s,i}-T_{o}\right)\times A_{i}}{H_{c,i}},$$

where *A*—area of the whole manikin surface (m^2^).

Total insulation for a body zone group (*I*_T,z,i_, m^2^K/W):

(3) $$I_{T,z,i}=\frac{\left(\sum (T_{s,i}\times A_{i})/\sum A_{z,i}^{n}\right)-T_{o})\times \sum A_{z,i}^{n}}{\sum H_{c,z,i}^{n}},$$

where $\sum A_{z,i}^{n}$ —area of all manikin zones included in the group (m^2^); $\sum H_{c,z,i}^{n}$ —dry heat loss from all manikin zones included in the group (W).

Total insulation for the whole body (*I*_T_, m^2^K/W):

(4) $$I_{T}=\frac{\left(\sum (T_{s,i}\times A_{i})/A\right)-T_{o})\times A}{\sum H_{c,i}}.$$

Basic (intrinsic) clothing insulation of an item (*I*_clu_, m^2^K/W) or an ensemble (*I*_cl_, m^2^K/W):

(5) $$I_{\mathrm{clu}}=I_{\mathrm{cl}}=I_{T}-\frac{I_{a}}{f_{\mathrm{cl}}},$$

where *I*_a_—air layer insulation (measured on the nude manikin, m^2^K/W); *f*_cl_—clothing area factor (n.d.).

Water vapour pressure at manikin surface temperature (Pa):

(6) $$p_{s}=e^{18.956-\frac{4030}{T_{s}+235}}\times RH_{\mathrm{sk}},$$

where *T*_s_—mean surface temperature of a manikin (°C); *RH*_sk_—relative humidity at manikin (textile) skin temperature (%).

Manikin surface temperature (°C):

(7) $$T_{s}=\sum \frac{T_{s,i}\times A_{i}}{A}.$$

Water vapour pressure at textile skin temperature (Pa):

(8) $$p_{\mathrm{sk}}=e^{18.956-\frac{4030}{T_{\mathrm{sk}}+235}}\times RH_{\mathrm{sk}},$$

where *T*_sk_—mean (textile) skin temperature of a manikin (°C), and where mean *RH*_sk_ over the various conditions irrespective of the clothing combination was 96%.

(9) $$T_{\mathrm{sk}}=\sum \frac{T_{\mathrm{sk},i}\times A_{i}}{A},$$

where *T*_sk,i_—mean (textile) skin temperature of a manikin zone *i* (°C).

Correction of the manikin skin temperature [98,100] (depends on “skin” properties):

(10) $$T_{\mathrm{sk},i}=T_{s,i}-0.0132\times Q_{i},$$

where *Q*_i_—heat flux from a manikin zone *i* (W/m^2^).

Water vapour pressure in the air (Pa):

(11) $$p_{a}=e^{18.956-\frac{4030}{T_{a}+235}}\times RH_{a},$$

where *T*_a_—air temperature (°C); *RH*_a_—relative air humidity (%).

Total evaporative resistance of a zone (m^2^Pa/W):

(12) $$R_{\mathrm{et},i}=\frac{\left(p_{\mathrm{sk},i}-p_{a}\right)\times A_{i}}{H_{i}},$$

where *p*_sk,i_—water vapour pressure at (textile) skin of a manikin zone *i* (Pa); H_i_—heat loss from a manikin zone *i* (W).

Heat flux (W/m^2^):

(13) $$Q=\frac{\sum H_{i}}{\sum A_{i}}.$$

Uncorrected evaporative resistance (m^2^Pa/W):

(14) $$R_{\mathrm{et},\mathrm{raw}}=\frac{\left(p_{s}-p_{a}\right)}{\sum \left(H_{e,i}\times \frac{A_{i}}{A}\right)},$$

where *H*_e,i_—assumed evaporative heat loss from a manikin zone *i* (W).

The assumption here is that conductive, convective and radiative heat exchange is close to zero, and the test is carried out at the isothermal conditions (*T*_a_ = *T*_s_ = *T*_sk_), i.e., the only heat exchange pathway is evaporation.

Total evaporative resistance corrected for water vapour pressure at skin temperature (m^2^Pa/W):

(15) $$R_{\mathrm{et}}=\frac{\left(p_{\mathrm{sk}}-p_{a}\right)}{\sum \left(H_{e,i}\times \frac{A_{i}}{A}\right)},$$

Clothing evaporative resistance (m^2^Pa/W):

(16) $$R_{\mathrm{ecl}}=R_{\mathrm{et}}-\frac{R_{\mathrm{ea}}}{f_{\mathrm{cl}}},$$

where *R*_ea_—air layer evaporative resistance (measured with textile skin, m^2^Pa/W).

Moisture permeability index (n.d.):

(17) $$i_{m}=\frac{I_{T}}{R_{\mathrm{et}}\times L},$$

where *L*—Lewis relation (16.5 × 10^−3^ K/Pa).

Clothing moisture permeability index (n.d.):

(18) $$i_{m,\mathrm{cl}}=\frac{I_{\mathrm{cl}}}{R_{\mathrm{ecl}}\times L}.$$

Relationship between clo and m^2^K/W:


1 clo = 0.155 m^2^K/W. (19)

### 2.7. Clothing

In total, 37 individual items or their variations (see Table 1 for images and description) and 25 clothing ensembles were measured including 150 dry measurements for thermal insulation and 36 wet measurements for evaporative resistance of 12 selected ensembles (see Table 2 for description and Table 3 for images). Each item and combination was tested at least twice. If the difference of two measurements was above 4%, then the measuring settings were checked, and additional measurements were carried out. In addition, the tested items and clothing combinations were photographed from front and side for documentation purposes and for clothing area factor (*f*_cl_) calculations. All the items were weighed.

## 3. Results and Discussion

The specifications and the insulation measurement results for the individual items (*I*_T_, *I*_clu_) are shown in Table 1. Figure A1 in Appendix C visualizes the individual items’ basic insulation ranked by the item group and/or body coverage region, e.g., feet, hands, head, underwear, shirts, jackets and their variations, etc. Table 2 presents the thermal insulation of the tested clothing ensembles (*I*_T_, *I*_cl_), the evaporative resistances (*R*_et,raw_, *R*_et_, *R*_et,cl_) and permeability indices (*i*_m_, *i*_m,cl_) of the selected clothing ensembles. In addition, the measured clothing area factors (*f*_cl_) for individual items and the clothing ensembles are given in Table 1 and Table 2, respectively. Table 3 presents the pictures of the clothing ensembles. Table A1 of Appendix A shows the thermal insulation and Table A2 of Appendix B the evaporative resistance values of the individual body zones and body regions.

### 3.1. Relationship between Measured and Summed Basic Clothing Insulation (I_cl_)

**Figure 1:**
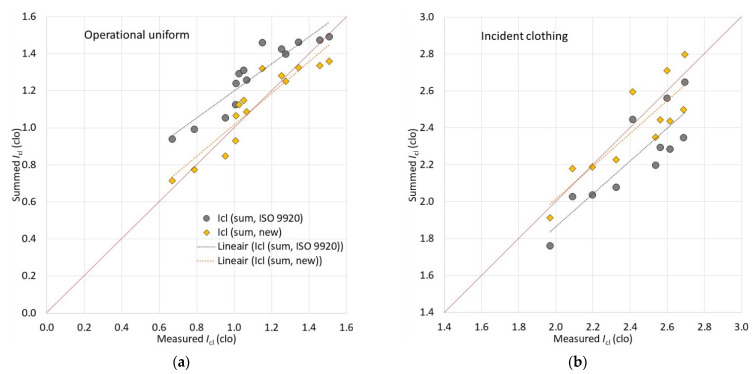
Separate relationships between measured and summed basic clothing insulation (*I*_cl_, clo) for (**a**) operational uniform and (**b**) incident clothing ensembles. Icl (sum, ISO 9920) represents summation according to ISO 9920, and Icl (sum, new) modified the summation for these datasets.

If the standard method for summing up the individual clothing items’ insulation [50] fit practically perfectly for the ambulance clothing system [50,70], then the firefighter clothing system tested in the present study differed clearly in this respect, creating two groups. For the operational uniform (OU) ensembles, the standard summation method overestimated the actual insulation (Figure 1a), and for the incident clothing systems, it underestimated the insulation (Figure 1b). It may be expected that the basic clothing insulation range between 1.4 and 2 clo can be predicted well; however, there were no ensembles in the range from 1.6 to 1.9 clo. When these OU and incident clothing groups were separated, then for clothing ensembles that covered incident scenarios, it was enough to adjust the intercept of the standard equation to acquire a reasonable prediction (Equation (20), Figure 1b).

(20) $$I_{\mathrm{cl}}=0.311+0.835\times \sum I_{\mathrm{clu}}.$$

When the OU ensembles are above 1.3–1.4 clo, the prediction (summation) may be considered as a reasonable match (Figure 1); then, for lighter clothing ensembles, the overestimation by summation is bigger. For OU ensembles, a better matching relationship had to be developed (R^2^ = 0.993, Equation (21), Figure 1a):

(21) $$I_{\mathrm{cl}}=0.975\times \sum I_{\mathrm{clu}}-0.194.$$

### 3.2. Clothing Surface Area Factor (f_cl_)

**Figure 2:**
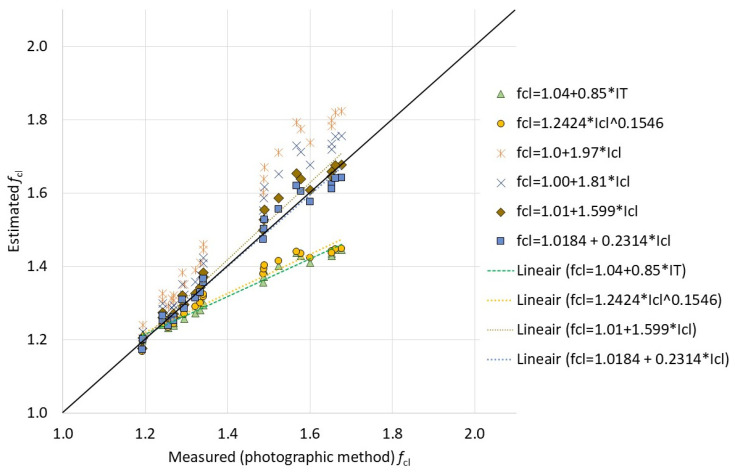
Relationship between the measured and the estimated clothing area factors for the firefighter clothing system by various available equations. *f*_cl_ (or fcl)—clothing area factor; IT—total clothing insulation; Icl—basic clothing insulation.

As in an earlier study on ambulance clothing [71], for firefighter clothing systems, a wide variety of available equations for *f*_cl_ calculation were utilized. The best estimations were not the ones derived specifically for a dedicated clothing system based on a layer-by-layer approach [71] for a winter clothing system [103] or estimation equations based on a wider database [50], but instead according to the recent estimation for a modern Western clothing database [63] (R^2^ = 0.978, Figure 2), possibly indicating similar trends in fashion:

(22) $$f_{\mathrm{cl}}=1.01+1.599\times I_{\mathrm{cl}}.$$

The specific equation for clothing area factor estimation (R^2^ = 0.978; *I*_cl_ in clo) for this clothing system was

(23) $$f_{\mathrm{cl}}=1.02+0.2314\times I_{\mathrm{cl}}.$$

Any further tests with other firefighter clothing ensembles may need to prove if the equations above also fit in general with those. Those combinations with the respiratory protective system (SCBA) did not specifically stand out from the general variation, neither for clothing area factor estimation nor for the basic clothing insulation summation method.

### 3.3. The Use of Collected Data

The outcome of the individual items and garment ensembles measurements allows us to evaluate the effects of (a) variation in individual items and their combinations, (b) ways of dressing, and (c) design solutions. Gathering specific basic data on firefighter clothing systems allowed us to adjust the clothing summation method for improved prediction accuracy when selecting and recommending the clothing combinations. The basic data on clothing evaporative resistance give us improved prediction accuracy when selecting and recommending the clothing combinations. In combination with input from other relevant research in this area [26,37,42,43,104,105,106,107], the collected data allow us to model the exposure to a variety of incident scenarios in the presently available clothing systems, and most importantly, validate those available models for rescue services incident scenarios and exposure conditions if the experimental data with the same or similar clothing systems will be available.

As insulation and evaporative resistance values of all individual body parts and body areas are also available (see Appendix A and Appendix B), there is a possibility to (a) use these datasets in advanced thermo-physiological models to obtain more detailed predictions; (b) find weak areas in the clothing systems and suggest improvements in clothing design; (c) perform more accurate exposure predictions for improved purchase support. However, experimental data have to be collected for proper model selection and validation. Knowing the clothing properties will greatly assist in decision making in relation to extreme weather events and indoor exposures [23,24]. In the same way, exposure to indoor (office) environments can be estimated according to ISO 7730 [82]. This standard introduces PMV (predicted mean vote) and PPD (predicted percentage of dissatisfied) indexes that relate the environmental parameters to thermal responses of the exposed population. However, the tools are not easily available for much more extreme exposure conditions that the firefighters face.

## 4. Conclusions and Future Perspectives

The work of this paper aimed to evaluate the thermal properties of the presently used firefighter clothing systems. Some complementary evaluations should be performed further on, for example: measuring body motion (walking) effects on manikins and the effects of higher air velocity and the combinations of these factors to estimate ventilation effects in the clothing systems. A separate line of research on firefighter clothing systems is the continued information collection and material testing for the effects of heat radiation levels.

Additionally, some common ways of wearing presently used garments should be tested, e.g., front zip open/closed/partly closed, Velcro or rubber bands at wrists or ankles tightened or loose, in order to collect more information on clothing performance for future design solution selection. In addition, the effects and usefulness of available cooling solutions [108] should be evaluated for firefighter use, taking into account logistical challenges. Further, testing of specific design solutions/ideas, including the effects of smart functions, must be considered in order to support the development of standards that are able to cover evaluation and certification needs of the new technical solutions so that they can safely be taken into use by the rescue services [109,110].

As many simple, but also advanced, thermo-physiological models do not support exposure to extreme environmental conditions or highly protective clothing (including impermeable layers), after checking the capacities of existing advanced models [40,44], the needs for improving the prediction models must be settled. Such validation requires considerable experimental work with human subjects. However, the use of such validated models allows us to evaluate clothing design solutions before and under prototyping phases and to diminish the need of later testing on human subjects and minimize costs on changes. At the same time, testing the protective clothing systems for compatibility and ergonomics is still needed [111,112].

The basic data on the present clothing system were collected, and the database was created. This allows us to proceed with:

- Selection and recommendations of clothing combinations for the best performance and comfort under specific exposure conditions;
- Evaluation of the design aspects of the protective clothing and protective clothing systems;
- Modelling and prediction model validation, and the comparison of their deviation from human responses in realistic conditions with human subjects;
- Supporting the development of the firefighter’s modular protective clothing system of the future;
- Developing digital decision support tools for rescue services’ purposes;
- Creating educational and demonstration materials for optimal protection selection.

## Figures and Tables

**Table 1:** Clothing item specifications and their total insulation (*I*_T_), clothing area factor (*f*_cl_) and basic clothing insulation (*I*_clu_).

Nr.	Item and Their Variation Description	Photo	Brand, Model	Size, Version	Weight (Grams)	Materials (Notes)	*I*_T_ m^2^K/W	*f*_cl_ n.d.	*I*_clu_ m^2^K/W
AL	Nude manikin (air layer insulation—*I*_a_)	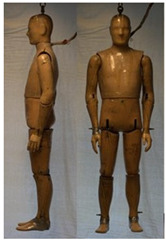					0.099	1.00	0.000
1	Socks	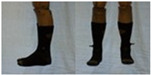	Groenendijk, Brandweer Nederland	43–45	62.2		0.107	1.01	0.009
2	Low safety shoes	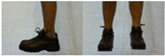	Neskrid, Crow S3 low	45, W11	1554.1	PU sole, leather uppers, fabric padding	0.107	1.02	0.010
3	High safety shoes	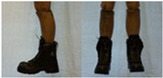	Haix, Airpower XR1	45	2153.4	PU sole, leather uppers, fabric padding	0.109	1.06	0.015
4	Firefighter boots	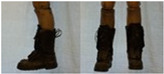	Haix, Fire flash gamma	44, W12	2939.3	PU sole, leather uppers, fabric padding, Gore-Crosstech	0.113	1.07	0.021
5	Baseball cap	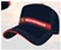	Groenendijk, Brandweer Nederland	one size	91.3	50% cotton 48% polyester 2% elastane	0.103	1.01	0.005
6	Beanie	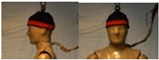	Groenendijk, Brandweer Nederland	one size	49.5	80% Wool 20% Acrylic	0.104	1.00	0.005
7	Balaclava/fire hood	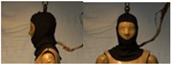	Viking Life Saving Equipment A/S, Type 1	one size	119.4	100% Aramid	0.107	1.01	0.009
8	Boxershorts (underwear)	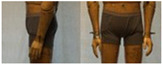	GS Sport	XL	69.4	95% Cotton 5% Elastane	0.106	1.01	0.008
9	T-shirt (underwear)	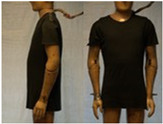	Narkonteks, Brandweer Nederland	L, regular	182.1	60% Mode Acrylic 40% Cotton, Ribana, 190 g/m^2^	0.124	1.03	0.027
10	Belt (stretch-heavy)	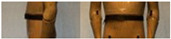	Groenendijk	105	106.2		0.102	1.00	0.003
11	Polo shirt, short sleeves	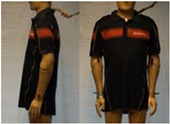	Narkonteks, Brandweer Nederland	L, regular	345.8	60% Mode Acrylic 40% Cotton, Pique, 200 g/m^2^	0.131	1.08	0.040
12	Polo shirt, long sleeves	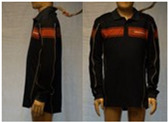	Narkonteks, Brandweer Nederland	L, regular	427.2	60% Mode Acrylic 40% Cotton, Pique, 200 g/m^2^	0.140	1.09	0.049
13	(Polo)sweater (400 gr)	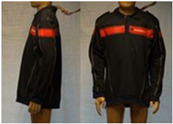	Narkonteks, Brandweer Nederland	L, regular	842.1	50% Mode Acrylic 50% Cotton, three thread fleece, 400 g/m^2^	0.148	1.14	0.061
14	Softshell (zip open at neck)	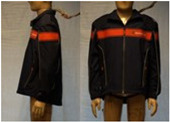	SIOEN, Brandweer Nederland	L, regular	787.2		0.145	1.11	0.056
14A	Softshell (fully zipped)	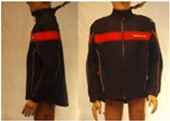	SIOEN, Brandweer Nederland	L, regular	787.2		0.146	1.14	0.059
15	Working trousers	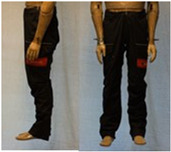	SIOEN, Brandweer Nederland	52, regular	697.7	42% modacrylic, 29% cotton 19% polyamide FR, 5% aramid 4% elastolefin, 1% antistatic (AST) yarn	0.136	1.12	0.047
16	Safety gloves (cut resistant) for technical rescue	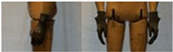	GUIDE, 313	11	82.7		0.102	1.01	0.004
17	Safety gloves (for structural firefighting)	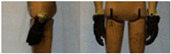	Eska, Helios E	10	366.2		0.102	1.03	0.005
18	Technical rescue jacket	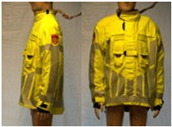	SIOEN, 233 Proviz/AS	L, regular	1431.7	OL: 54% Nomex^®^, 28% polyester 17% viscose FR, 1% AST Lining: 100% aramid laminated with bi-component ePTFE membrane + AST	0.162	1.17	0.077
19	Firefighter top layer jacket (to be used on top of item 18)	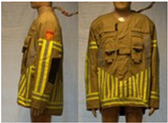	SIOEN, TL TWIN/AS	L, regular	1312.0	OL: 100% aramid + AST Lining: 50% aramid 50% viscose FR	0.171	1.17	0.087
21	Firefighter jacket (corresponds to EN 469)	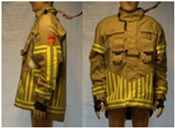	SIOEN, 830 Twin/AS	L, regular	1793.8	OL: 100% aramid + AST ML: 100% aramid laminated with bi-component ePTFE membrane Lining: 100% aramid + AST	0.177	1.23	0.097
22	Firefighter trousers, standard model (corresponds to EN 469)	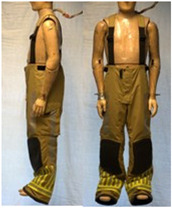	SIOEN	L, regular	1712.5	OL: 100% aramid + AST ML: 100% aramid laminated with bi-component ePTFE membrane Lining: 100% aramid + AST	0.168	1.32	0.093
23	Firefighter trousers, dungarees (corresponds to EN 469)	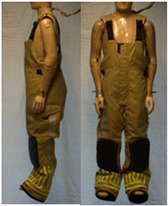	SIOEN	L, regular	1795.3	OL: 100% aramid + AST ML: 100% aramid laminated with bi-component ePTFE membrane Lining: 100% aramid + AST	0.167	1.36	0.095
24	Firefighter coverall (corresponds to EN 469)	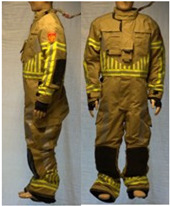	SIOEN	L, regular	2883.2	OL: 100% aramid + AST ML: 100% aramid laminated with bi-component ePTFE membrane Lining: 100% aramid + AST	0.303	1.47	0.236
25	Firefighter helmet	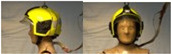	Dräger, HPS 7000 H1	50–60	1621.3		0.103	1.07	0.011
26	Face and neck cover (hollanddoek, connected to helmet (item 25), closed in front)	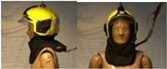	Dräger	one size	161.3		0.107	1.12	0.018
26A	Face and neck cover (hollanddoek, connected to helmet (item 25), open in front, fixed at back side)	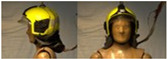	Dräger	one size	161.3		0.105	1.10	0.016
27	Technical rescue helmet	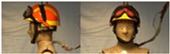	MSA Gallet, F2 X-trem	52–64 cm	830.1		0.102	1.05	0.008
28	Self-Contained Breathing Apparatus (SCBA, bottles only)	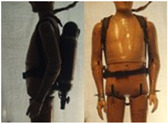	Interspiro, V-RWS	XXL	11,469.9	Composite with 2 bottles.	0.104	1.15	0.018
29	High visibility vest	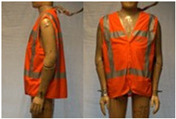	Coprit, V-RWS	XXL	166.4	100% polyester	0.117	1.08	0.026
30	Winter jacket (doorwerkjas) with thermal lining	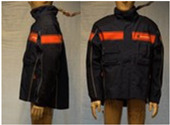	SIOEN, Brandweer Nederland	L, regular	1594.3	OL and lining: 100% Polyester Membrane: 100% Polyurethane Thermal liner: 100% Polyester insulation between 100% polyamide layers	0.163	1.19	0.080
30A	Winter jacket (doorwerkjas) without thermal lining (outer shell only)	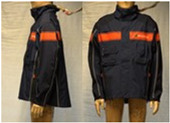	SIOEN, Brandweer Nederland	L, regular	1253.1	OL and lining: 100% Polyester Membrane: 100% Polyurethane	0.151	1.16	0.066
30B	Winter jacket (doorwerkjas) without thermal lining (outer shell only) and with hood half way (it was not allowed to cut)	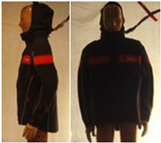	SIOEN, Brandweer Nederland	L, regular	1353.0	OL and lining: 100% Polyester Membrane: 100% Polyurethane	0.161	1.19	0.078
30C	Thermal liner of the winter jacket alone	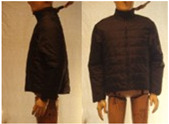	SIOEN, Brandweer Nederland	L, regular	341.2	100% Polyester insulation between 100% polyamide layers	0.156	1.13	0.068
31	SCBA system (items 26, 28 and facemask from Dräger fitting the helmet)	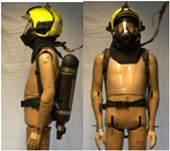	Dräger, FPS 7000/Interspiro	M, HMC	755.0	Rubber/plastic glass	0.113	1.26	0.034
31A	Face mask	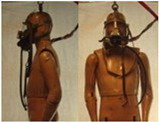	Interspiro		1228.7	Rubber/plastic glass	0.100	1.07	0.008
31B	SCBA system (items 28 and 31A)	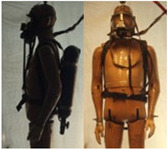	Interspiro		12,698.6	Rubber/plastic glass, composite	0.106	1.22	0.025

**Table 2:** Clothing ensembles and their properties * (for images of the clothing ensembles see Table 3).

Code	Items in the Ensemble (See Table 1)	*I*_T_ m^2^K/W	*f*_cl_ n.d.	*I*_cl_ m^2^K/W	*I*_cl_ clo	*R*_et,raw_ m^2^Pa/W	*R*_et_ m^2^Pa/W	*R*_ecl_ m^2^Pa/W	*i*_m_ n.d.	*i*_m,cl_ n.d.
AL	Nude manikin (air layer insulation—*I*_a_)	0.099	1.00	0.000	0.00					
SK	Manikin’s textile skin (cotton)	0.131	1.03	0.040	0.26	11.5	8.0			
C1	1, 2, 8, 9, 10, 11, 15	0.187	1.19	0.104	0.67	22.3	18.7	12.0	0.61	0.52
C1A	1, 2, 8, 9, 10, 11, 14, 15	0.236	1.24	0.157	1.01	35.2	31.5	25.0	0.45	0.37
C1B	1, 2, 8, 9, 10, 11, 14, 15 The same as C1A but with softshell zip fully closed	0.245	1.24	0.166	1.07					
C2	1, 2, 8, 9, 10, 12, 15	0.205	1.19	0.122	0.79	25.7	22.0	15.4	0.56	0.48
C2A	1, 2, 8, 9, 10, 12, 14, 15	0.237	1.26	0.159	1.03	32.9	29.3	23.0	0.49	0.42
C2B	1, 2, 8, 9, 10, 12, 14, 15, 16	0.241	1.27	0.163	1.05					
C2C	1, 3, 8, 9, 10, 12, 14, 15, 29	0.255	1.29	0.179	1.15					
C2D	1, 3, 6, 8, 9, 10, 12, 15, 30A	0.273	1.32	0.198	1.28	45.5	41.8	35.7	0.40	0.33
C2E	1, 3, 6, 8, 9, 10, 12, 15, 30B	0.282	1.33	0.208	1.34					
C2F	1, 3, 6, 8, 9, 10, 12, 15, 30	0.300	1.34	0.226	1.46					
C2G	1, 3, 6, 8, 9, 10, 12, 15, 16, 30 The same as C2F but with technical rescue gloves (Table 1, item 16)	0.307	1.34	0.234	1.51	48.0	44.3	38.3	0.42	0.37
C3	1, 2, 8, 9, 10, 13, 15	0.227	1.26	0.148	0.95					
C3A	1, 3, 6, 8, 9, 10, 13, 15, 16	0.234	1.27	0.156	1.01					
C3B	1, 3, 6, 8, 9, 10, 13, 14, 15, 16	0.271	1.29	0.194	1.25	41.9	38.2	32.0	0.43	0.36
C4	1, 4, 8, 9, 10, 12, 15, 17, 18, 19, 22, 31 (consisting of 26 (hollanddoek fixed to 25), 28 and facemask similar to 31A but fixed to helmet)	0.477	1.68	0.418	2.69	85.3	81.4	76.7	0.35	0.33
C5	1, 4, 8, 9, 10, 12, 15, 17, 24, 26 (as part of 25)	0.436	1.60	0.374	2.41					
C6	1, 4, 8, 9, 10, 12, 15, 17, 21, 22, 26 (as part of 25)	0.456	1.58	0.393	2.54	74.4	70.6	65.6	0.39	0.36
C6A	1, 4, 8, 9, 10, 12, 15, 17, 21, 22, 31 (consisting of 26 (hollanddoek fixed to 25), 28 and facemask similar to 31A but fixed to helmet)	0.465	1.65	0.405	2.62	87.4	83.6	78.8	0.34	0.31
C6B	1, 4, 7, 8, 9, 10, 12, 15, 17, 21, 22, 25, 28, 31A	0.476	1.66	0.417	2.69					
C6C	1, 4, 8, 9, 10, 12, 15, 17, 21, 23, 31 (consisting of 26 (hollanddoek fixed to 25), 28 and facemask similar to 31A but fixed to helmet)	0.457	1.65	0.397	2.56					
C7	1, 4, 8, 9, 10, 12, 15, 17, 18, 19, 22, 26 (hollanddoek fixed to 25)	0.466	1.57	0.403	2.60					
C8	1, 4, 8, 9, 10, 12, 15, 17, 18, 22, 26A (hollanddoek front open fixed to 25)	0.425	1.52	0.361	2.33	76.4	72.6	67.4	0.36	0.32
C9	1, 4, 8, 9, 10, 15, 16, 18, 22, 27	0.372	1.49	0.305	1.97					
C9A	1, 4, 8, 9, 10, 12, 15, 16, 18, 22, 27	0.390	1.49	0.324	2.09	63.2	59.4	54.0	0.40	0.36
C9B	1, 4, 8, 9, 10, 12, 15, 17, 18, 22, 27	0.407	1.49	0.341	2.20					

* *I*_T_—total clothing insulation; *f*_cl_—clothing area factor; *I*_cl_—basic clothing insulation; *R*_et,raw_—uncorrected total evaporative resistance; *R*_et_—corrected total evaporative resistance; *R*_ecl_—corrected clothing evaporative resistance; *i*_m_—moisture permeability index; *i*_m,cl_—clothing moisture permeability index; for details see Section 2.6.

**Table 3:** Images of the tested clothing ensembles (for properties and composition, see Table 2).

AL	SK		
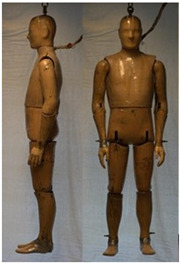	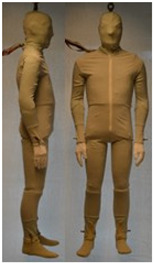		
C1	C1A	C2	C2A
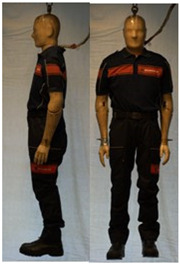	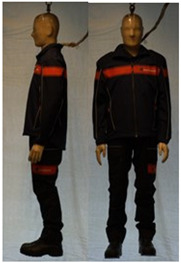	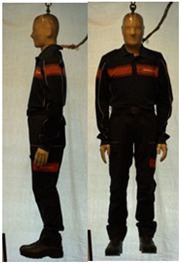	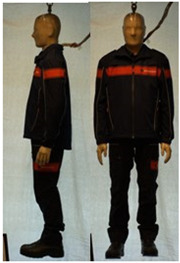
C2B	C2C	C2D	C2E
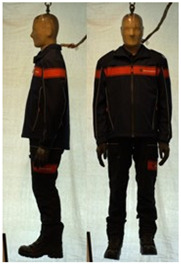	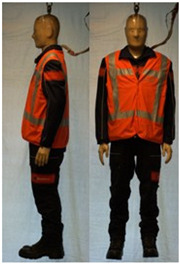	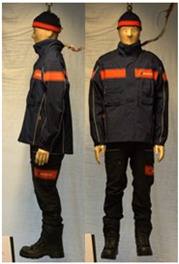	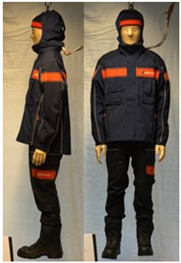
C2F	C3	C3A	C3B
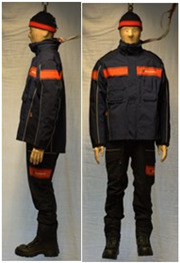	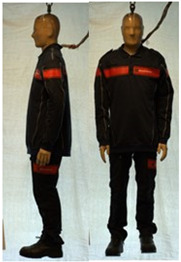	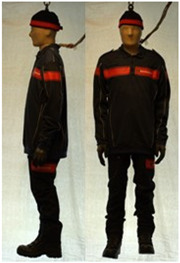	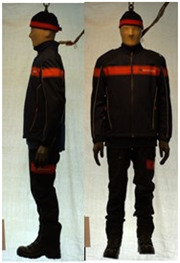
C4	C5	C6	C6A
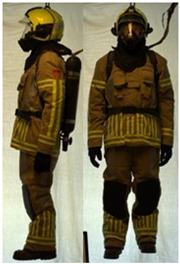	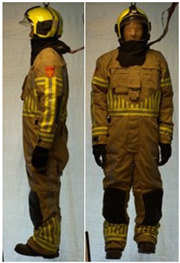	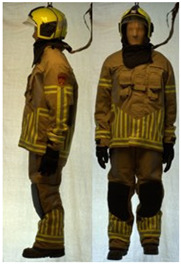	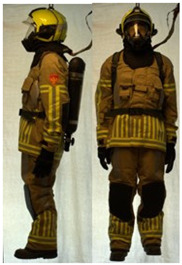
C6B	C6C	C7	C8
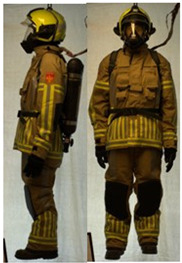	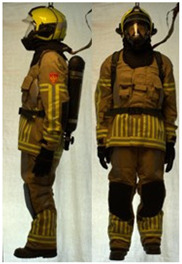	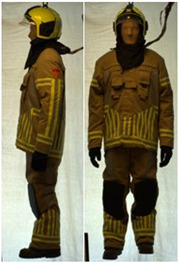	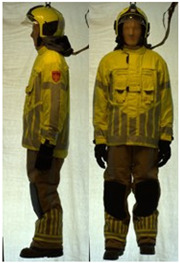
C9	C9A	C9B	
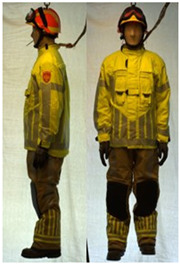	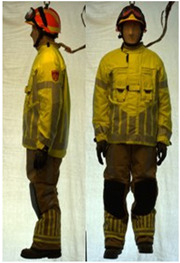	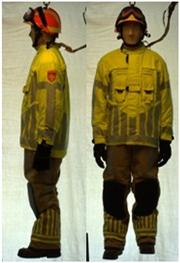	

## Data Availability

Not applicable.

## Appendix A

**Table A1:** Clothing ensembles’ local and regional clothing insulation values (*I*_T,i_, *I*_T,z,i_, m^2^K/W).

Code	Head	Chest	Back	Abdomen	Buttocks	Torso	Upper Arms	Lower Arms	Arms	Hands	Thighs	Calves	Legs	Feet	Head, Hands and Feet Excluded
AL	0.106	0.113	0.097	0.095	0.076	0.097	0.119	0.105	0.113	0.101	0.097	0.082	0.090	0.108	0.097
C1	0.101	0.279	0.251	0.304	0.267	0.263	0.211	0.104	0.156	0.101	0.230	0.174	0.201	0.264	0.207
C1A	0.098	0.413	0.388	0.469	0.472	0.419	0.360	0.232	0.302	0.100	0.255	0.186	0.222	0.268	0.294
C1B	0.105	0.451	0.405	0.520	0.498	0.448	0.368	0.244	0.312	0.103	0.260	0.191	0.227	0.270	0.305
C2	0.101	0.238	0.214	0.329	0.285	0.246	0.278	0.205	0.247	0.117	0.246	0.179	0.214	0.267	0.233
C2A	0.099	0.387	0.347	0.531	0.438	0.397	0.385	0.278	0.337	0.110	0.254	0.175	0.215	0.266	0.292
C2B	0.098	0.389	0.357	0.526	0.455	0.404	0.394	0.287	0.345	0.122	0.253	0.174	0.214	0.273	0.294
C2C	0.101	0.456	0.425	0.639	0.574	0.480	0.411	0.295	0.360	0.112	0.245	0.208	0.228	0.285	0.321
C2D	0.155	0.468	0.342	0.597	0.500	0.431	0.435	0.338	0.391	0.123	0.248	0.206	0.228	0.282	0.317
C2E	0.211	0.461	0.340	0.597	0.496	0.427	0.439	0.338	0.394	0.124	0.248	0.205	0.228	0.284	0.316
C2F	0.166	0.581	0.508	0.680	0.680	0.577	0.602	0.484	0.550	0.121	0.242	0.209	0.226	0.274	0.361
C2G	0.157	0.626	0.554	0.742	0.702	0.623	0.624	0.489	0.560	0.136	0.245	0.206	0.227	0.284	0.370
C3	0.101	0.280	0.248	0.443	0.453	0.302	0.329	0.261	0.299	0.155	0.241	0.180	0.211	0.275	0.259
C3A	0.143	0.269	0.232	0.427	0.411	0.286	0.314	0.238	0.280	0.152	0.231	0.202	0.218	0.268	0.255
C3B	0.145	0.414	0.378	0.601	0.598	0.442	0.410	0.297	0.358	0.123	0.249	0.219	0.235	0.272	0.319
C4	0.276	0.706	0.609	0.764	0.601	0.660	0.526	0.414	0.480	0.259	0.618	0.434	0.528	0.354	0.560
C5	0.207	0.670	0.534	0.585	0.529	0.581	0.551	0.389	0.480	0.262	0.545	0.439	0.497	0.341	0.523
C6	0.207	0.730	0.529	0.793	0.764	0.657	0.578	0.419	0.506	0.259	0.602	0.421	0.514	0.345	0.560
C6A	0.281	0.644	0.542	0.730	0.622	0.614	0.522	0.392	0.466	0.264	0.613	0.425	0.521	0.348	0.539
C6B	0.315	0.654	0.520	0.722	0.608	0.603	0.520	0.402	0.470	0.280	0.631	0.432	0.533	0.352	0.542
C6C	0.272	0.642	0.549	0.720	0.579	0.607	0.500	0.413	0.462	0.258	0.597	0.418	0.510	0.342	0.531
C7	0.207	0.812	0.623	0.842	0.783	0.735	0.535	0.419	0.485	0.255	0.613	0.426	0.521	0.350	0.578
C8	0.198	0.626	0.454	0.717	0.734	0.576	0.471	0.387	0.437	0.250	0.585	0.424	0.507	0.344	0.513
C9	0.155	0.545	0.411	0.777	0.772	0.537	0.401	0.349	0.379	0.155	0.565	0.426	0.499	0.340	0.478
C9A	0.153	0.664	0.447	0.796	0.734	0.590	0.479	0.390	0.441	0.147	0.586	0.433	0.514	0.351	0.521
C9B	0.154	0.638	0.436	0.727	0.777	0.574	0.468	0.388	0.435	0.247	0.590	0.426	0.511	0.348	0.513

## Appendix B

**Table A2:** Selected clothing ensembles’ local and regional evaporative resistances (m^2^Pa/W, correction acc. to Wang et al., 2010).

Code	All	Head	Chest	Back	Abdomen	Buttocks	Torso	Upper Arms	Lower Arms	Arms	Hands	Thighs	Calves	Legs	Feet	Head, Hands and Feet Excluded	Hands and Feet Excluded	Hands Excluded
SK	8.0	8.0	9.5	9.5	6.0	9.7	8.7	9.5	7.6	8.7	5.3	6.7	10.3	7.8	6.4	8.4	8.3	8.2
C1	18.7	8.9	21.8	18.7	36.9	27.9	23.1	19.9	8.1	13.7	6.0	26.5	21.7	24.3	107.1	20.5	18.8	20.3
C1A	31.5	9.8	66.0	63.5	74.0	48.5	62.5	65.8	44.7	55.9	8.2	28.1	21.5	24.9	102.4	40.2	33.3	35.2
C2	22.0	9.6	24.6	18.5	39.3	29.3	24.2	26.5	17.7	22.8	9.0	28.6	21.2	25.0	106.7	24.2	21.8	23.5
C2A	29.3	8.9	60.0	67.1	78.6	44.0	61.3	66.9	53.9	61.7	6.2	23.6	22.7	23.1	97.3	38.9	31.8	33.6
C2D	41.8	16.8	161.9	112.1	184.7	53.6	112.8	94.3	85.6	89.6	10.8	27.0	27.9	27.0	113.2	51.9	45.0	47.3
C2G	44.3	17.9	179.2	129.9	151.9	64.6	124.8	124.1	94.4	110.7	14.6	23.2	30.5	25.3	105.8	52.8	46.2	48.5
C3B	38.2	13.7	87.4	83.5	118.9	54.2	82.6	71.6	50.9	60.4	11.9	30.0	27.7	28.7	87.4	47.1	40.1	42.0
C4	81.4	44.6	139.6	101.8	134.6	124.3	120.7	85.2	71.9	79.9	41.1	84.1	75.4	78.9	94.0	91.7	84.7	85.3
C6	70.6	22.8	114.1	77.2	133.3	100.6	98.0	88.7	77.9	84.3	46.1	79.8	73.4	76.0	96.7	85.3	71.0	72.5
C6A	83.6	48.7	115.6	112.8	134.1	121.3	117.9	89.7	77.4	84.7	42.7	82.8	76.7	78.5	111.4	92.2	86.0	87.6
C8	72.6	24.5	112.1	73.8	183.9	110.4	100.4	71.2	65.3	68.8	51.0	81.0	97.0	85.3	108.8	85.7	72.2	74.1
C9A	59.4	16.3	107.7	68.3	144.4	109.9	93.5	69.0	61.4	66.0	22.4	79.6	75.2	77.1	101.2	79.6	62.4	64.2
