# Correction: Kuklane et al. A Database of Static Thermal Insulation and Evaporative Resistance Values of Dutch Firefighter Clothing Items and Ensembles. *Biology* 2022, *11*, 1813

**DOI:** 10.3390/biology14010065

**Published:** 2025-01-14

**Authors:** Kalev Kuklane, Jakob Eggeling, Maurice Kemmeren, Ronald Heus

**Affiliations:** 1 Team Fire Service Science, Netherlands Academy of Crisis Management and Fire Service Science, Netherlands Institute for Public Safety, Zilverstraat 91, 2718 RP Zoetermeer, The Netherlands; 2 Division of Ergonomics and Aerosol Technology, Department of Design Sciences, Lund University, Box 118, 22100 Lund, Sweden; 3 Team COLS, Netherlands Institute for Public Safety, Zilverstraat 91, 2718 RP Zoetermeer, The Netherlands

## Error in Appendix A, Table A1

The original publication contained an erroneous data line in Appendix A, “Table A1. Clothing ensembles’ local and regional clothing insulation values (*I*_T,i,_ *I*_T,z,i_, m^2^K/W)” [1]. The data line for ensemble C4 was mistakenly the same as for the air layer (AL). The error occurred due to uncorrected linking to the individual test files. Complete Table A1 with correct data for ensemble C4 is presented below. The authors apologize for any inconvenience caused and state that the scientific conclusions are unaffected as the annex was only intended as supplementary material with local insulation data.

**Table A1:** Clothing ensembles’ local and regional clothing insulation values (*I*_T,i_, *I*_T,z,i_, m^2^K/W).

Code	Head	Chest	Back	Abdomen	Buttocks	Torso	Upper Arms	Lower Arms	Arms	Hands	Thighs	Calves	Legs	Feet	Head, Hands and Feet Excluded
AL	0.106	0.113	0.097	0.095	0.076	0.097	0.119	0.105	0.113	0.101	0.097	0.082	0.090	0.108	0.097
C1	0.101	0.279	0.251	0.304	0.267	0.263	0.211	0.104	0.156	0.101	0.230	0.174	0.201	0.264	0.207
C1A	0.098	0.413	0.388	0.469	0.472	0.419	0.360	0.232	0.302	0.100	0.255	0.186	0.222	0.268	0.294
C1B	0.105	0.451	0.405	0.520	0.498	0.448	0.368	0.244	0.312	0.103	0.260	0.191	0.227	0.270	0.305
C2	0.101	0.238	0.214	0.329	0.285	0.246	0.278	0.205	0.247	0.117	0.246	0.179	0.214	0.267	0.233
C2A	0.099	0.387	0.347	0.531	0.438	0.397	0.385	0.278	0.337	0.110	0.254	0.175	0.215	0.266	0.292
C2B	0.098	0.389	0.357	0.526	0.455	0.404	0.394	0.287	0.345	0.122	0.253	0.174	0.214	0.273	0.294
C2C	0.101	0.456	0.425	0.639	0.574	0.480	0.411	0.295	0.360	0.112	0.245	0.208	0.228	0.285	0.321
C2D	0.155	0.468	0.342	0.597	0.500	0.431	0.435	0.338	0.391	0.123	0.248	0.206	0.228	0.282	0.317
C2E	0.211	0.461	0.340	0.597	0.496	0.427	0.439	0.338	0.394	0.124	0.248	0.205	0.228	0.284	0.316
C2F	0.166	0.581	0.508	0.680	0.680	0.577	0.602	0.484	0.550	0.121	0.242	0.209	0.226	0.274	0.361
C2G	0.157	0.626	0.554	0.742	0.702	0.623	0.624	0.489	0.560	0.136	0.245	0.206	0.227	0.284	0.370
C3	0.101	0.280	0.248	0.443	0.453	0.302	0.329	0.261	0.299	0.155	0.241	0.180	0.211	0.275	0.259
C3A	0.143	0.269	0.232	0.427	0.411	0.286	0.314	0.238	0.280	0.152	0.231	0.202	0.218	0.268	0.255
C3B	0.145	0.414	0.378	0.601	0.598	0.442	0.410	0.297	0.358	0.123	0.249	0.219	0.235	0.272	0.319
C4	0.276	0.706	0.609	0.764	0.601	0.660	0.526	0.414	0.480	0.259	0.618	0.434	0.528	0.354	0.560
C5	0.207	0.670	0.534	0.585	0.529	0.581	0.551	0.389	0.480	0.262	0.545	0.439	0.497	0.341	0.523
C6	0.207	0.730	0.529	0.793	0.764	0.657	0.578	0.419	0.506	0.259	0.602	0.421	0.514	0.345	0.560
C6A	0.281	0.644	0.542	0.730	0.622	0.614	0.522	0.392	0.466	0.264	0.613	0.425	0.521	0.348	0.539
C6B	0.315	0.654	0.520	0.722	0.608	0.603	0.520	0.402	0.470	0.280	0.631	0.432	0.533	0.352	0.542
C6C	0.272	0.642	0.549	0.720	0.579	0.607	0.500	0.413	0.462	0.258	0.597	0.418	0.510	0.342	0.531
C7	0.207	0.812	0.623	0.842	0.783	0.735	0.535	0.419	0.485	0.255	0.613	0.426	0.521	0.350	0.578
C8	0.198	0.626	0.454	0.717	0.734	0.576	0.471	0.387	0.437	0.250	0.585	0.424	0.507	0.344	0.513
C9	0.155	0.545	0.411	0.777	0.772	0.537	0.401	0.349	0.379	0.155	0.565	0.426	0.499	0.340	0.478
C9A	0.153	0.664	0.447	0.796	0.734	0.590	0.479	0.390	0.441	0.147	0.586	0.433	0.514	0.351	0.521
C9B	0.154	0.638	0.436	0.727	0.777	0.574	0.468	0.388	0.435	0.247	0.590	0.426	0.511	0.348	0.513
